# Supplementary material for: Mitochondrial Permeability Transition Causes Mitochondrial Reactive Oxygen Species- and Caspase 3-Dependent Atrophy of Single Adult Mouse Skeletal Muscle Fibers
Source: Cells. 2021 Sep 29;10(10):2586. doi: 10.3390/cells10102586 (PMC8534155; doi:10.3390/cells10102586)
Supplement: Supplementary file 1 [file cells-10-02586-s001.zip › cells-1363824-supplementary.pdf]

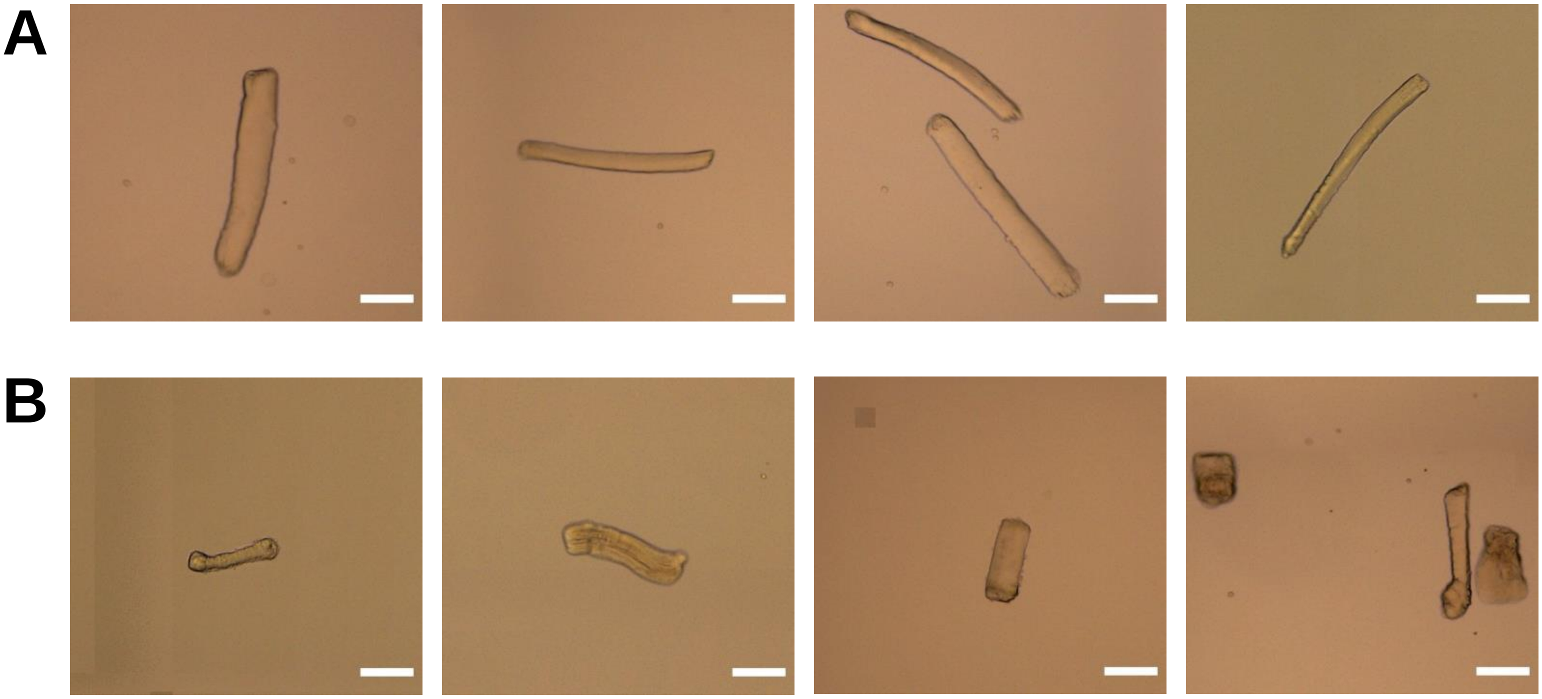

Figure S1. Representative images of (A) normal isolated muscle fibers and (B) hypercontracted muscle fibers in culture following isolation from adult mouse FDB muscles. Scale bar represents 100 µm.

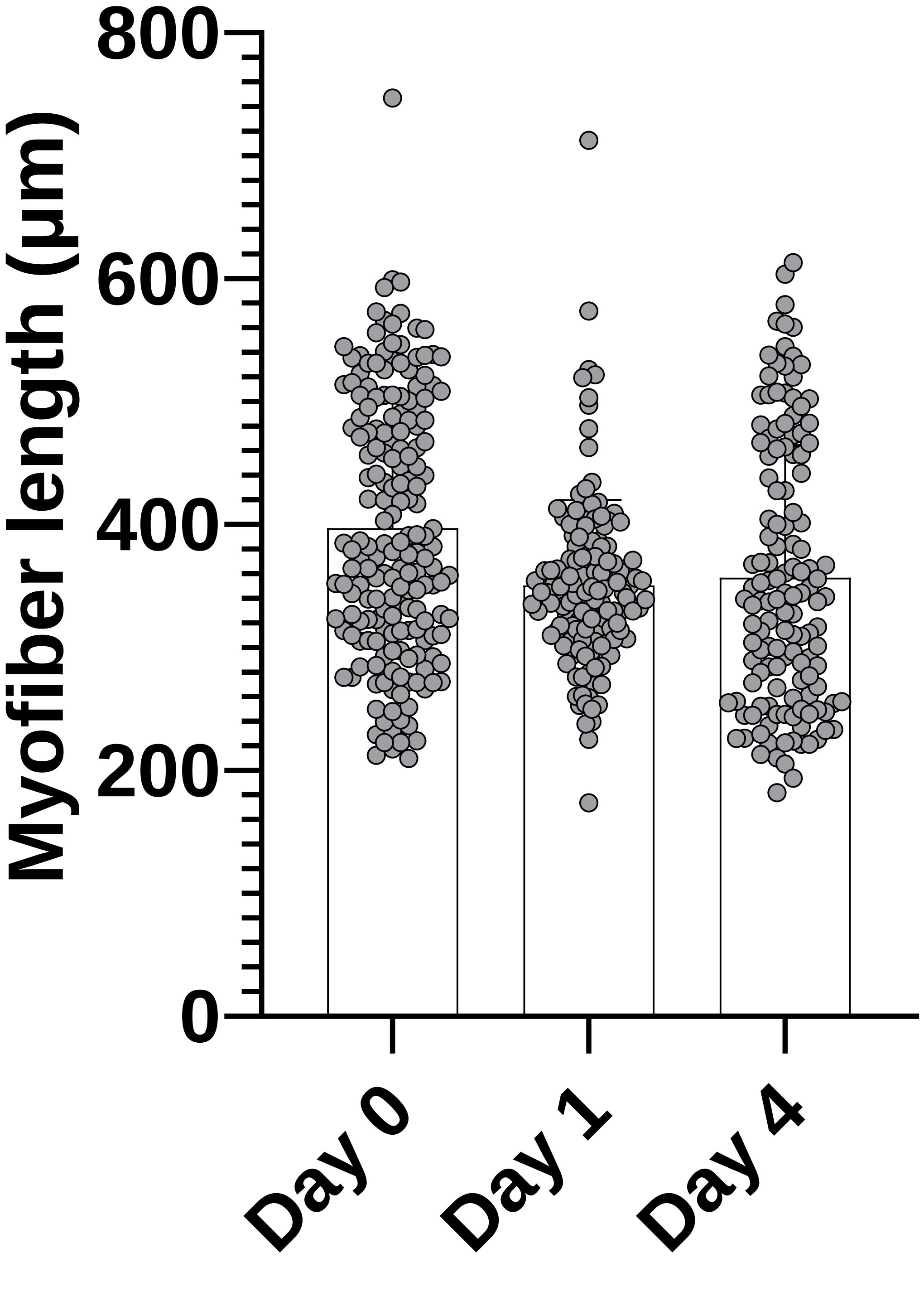

Figure S2. To ensure change in myofiber length did not bias myofiber diameter measurements, length of single living myofibers was assessed at baseline, after 1 day, and after 4 days in culture conditions. No significant change in length occurred between Day 0 and Day 1 or Day 4, indicating myofiber diameter measurements were unlikely to be impacted by change in myofiber length.

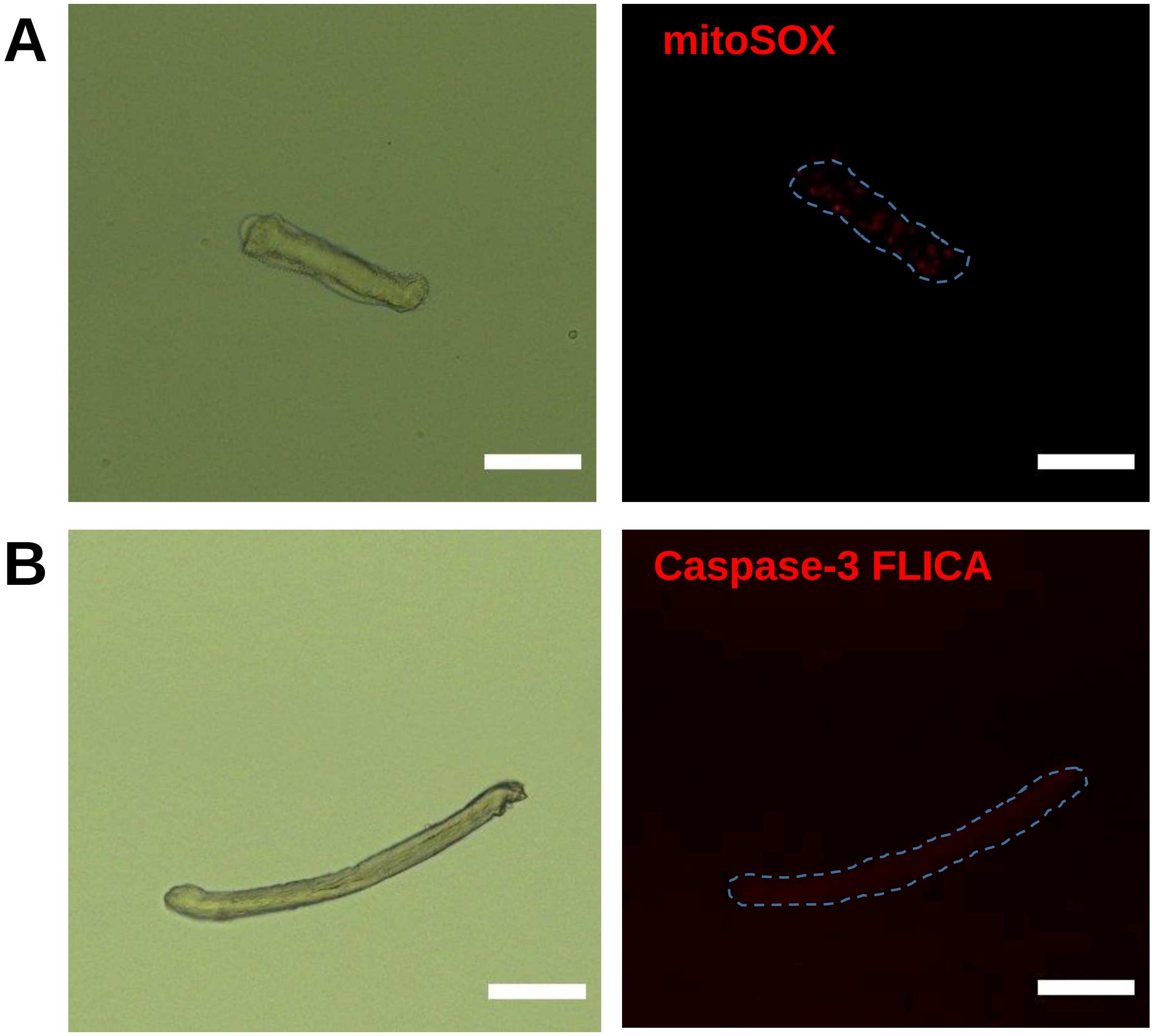

Figure S3. Representative images of baseline (A) mitoSOX and (B) caspase-3 FLICA fluorescence immediately following isolation in single mouse FDB muscle fibers. Contrast of the fluorescent images shown here has been enhanced to allow for visual identification of signal, but actual analysis was carried out on the raw, unenhanced fluorescent signal. Dashed lines indicate outline of muscle fiber. Scale bar represents 100 µm.

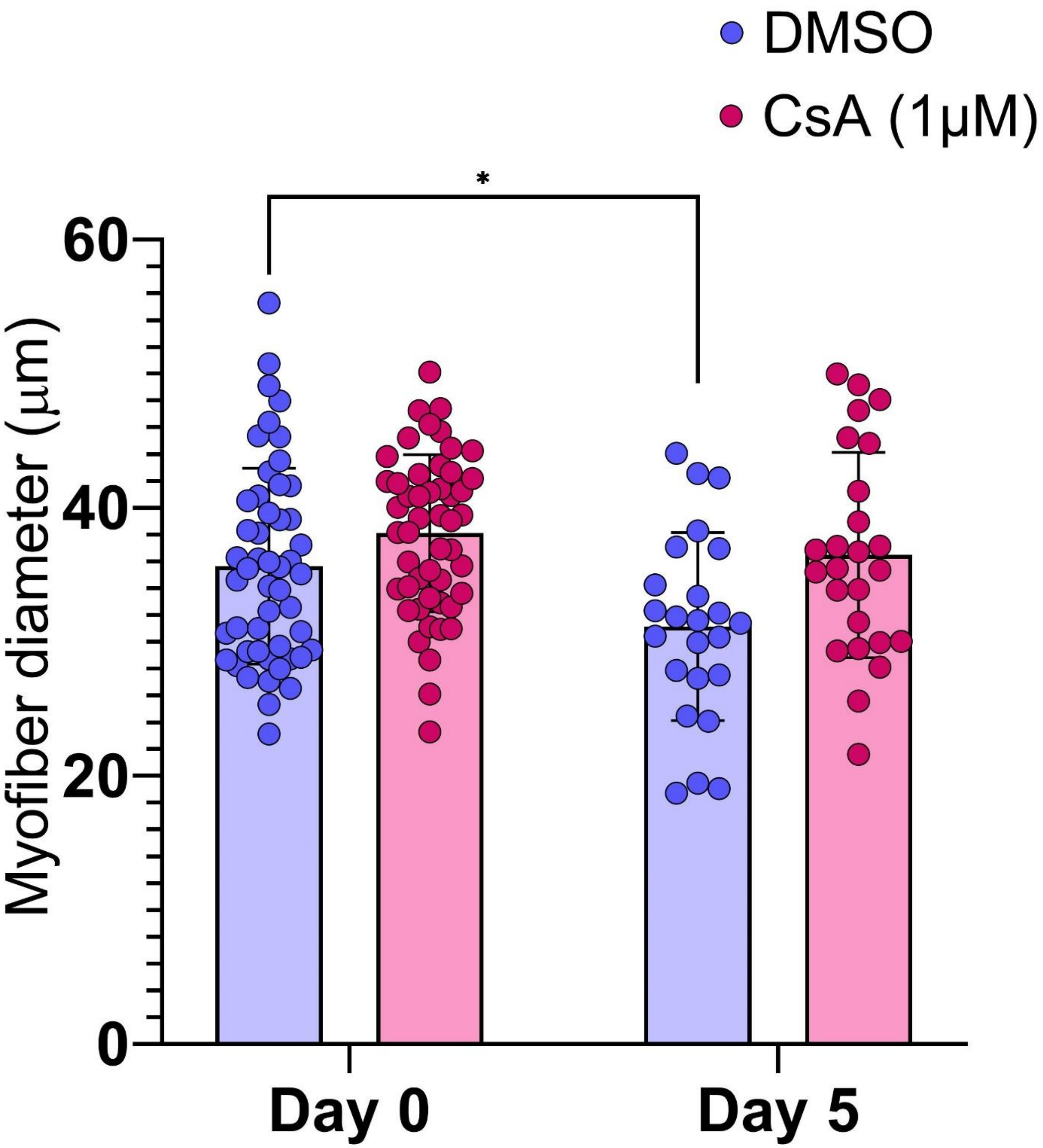

Figure S4. To determine if inhibition of MPT prevents atrophy in a single muscle fiber model of disuse, single mouse FDB myofibers were kept in culture conditions and treated with DMSO (vehicle control) or 1 µM cyclosporine A (CsA; cyclophilin D (CypD)-dependent inhibitor of MPT) for 5 days, and myofiber diameter was assessed pre- and post-treatment. Inhibition of MPT prevented a reduction in myofiber diameter over the 5-day period. *$P<0.05$ versus Day 0.
